# The Influence of Muscular Strength and Local Muscular Endurance on Accuracy of Estimated Repetitions to Failure in Resistance-Trained Males

**DOI:** 10.3390/sports10020027

**Published:** 2022-02-21

**Authors:** Daniel A. Hackett, Angelo Sabag

**Affiliations:** 1 Physical Activity, Lifestyle, Ageing and Wellbeing Faculty Research Group, School of Health Sciences, Faculty of Medicine and Health, The University of Sydney, Camperdown, NSW 2006, Australia; 2 NICM Health Research Institute, Western Sydney University, Westmead, NSW 2145, Australia; a.sabag@westernsydney.edu.au

**Keywords:** resistance training, repetition maximum, training intensity, weightlifting

## Abstract

This study investigated whether muscular strength and local muscular endurance (LME) influences accuracy of estimated repetitions to failure (ERF) during resistance exercise. Twenty resistance-trained males (age 26.3 ± 6.9 years) completed five sets of 10 repetitions at 70% 1RM for the bench press and squat. Following the 10th repetition of each set, participants reported ERF and then continued to concentric failure. Participants were separated into one of two groups based on muscular strength and LME. There were no significant differences between strength groups for error in ERF, ERF, and actual repetitions to failure (ARF). High-LME compared to Low-LME had greater ERF for all sets of bench press (*p* < 0.05) and two sets of squat (*p* < 0.05). Greater ARF was observed in High-LME for two of five sets for bench press (*p* < 0.05) and squat (*p* < 0.05). High-LME had greater error in ERF for bench press set 1 (*p* < 0.01) and set 4 (*p* = 0.04), while for set 1 only for squat (*p* = 0.01). Findings indicate that LME influences accuracy of ERF during the initial set of bench press and squat as well as a latter set for the bench press. Future studies with larger sample sizes are warranted to explore whether LME affects accuracy of ERF across multiple sets.

## 1. Introduction

Estimated repetitions to failure (ERF) is a resistance training tool that can be used to monitor and prescribe resistance exercise and is also referred to as repetitions in reserve (RIR) [1,2]. The accuracy of ERF is predominantly dependent on the proximity to concentric failure with greater accuracy when ERF is reported closer to failure [3,4]. We previously showed that error in ERF was > 2 repetitions when there were 7–10 actual repetitions to failure (ARF) compared to an error in ERF of ~1 repetition when there was 0–5 ARF [3]. It has been proposed that lifters may rely upon exertional sensations to guide their ERF, some of which include muscle activation, afferent signals from muscle proprioceptors, and mechanoreceptors [1]. Furthermore, since there is greater exertion and decreases in mean concentric velocity (MCV) as concentric failure is approached within a set, it appears likely that these responses play an important role towards increasing the accuracy of ERF [5,6]. Two other factors that are shown to influence the accuracy of ERF include resistance training experience and sex differences [3,7,8]. Greater accuracy in ERF was observed among experienced compared to novice resistance trainers during squats [8]. Additionally, females compared to males were found to be less accurate with reporting ERF during the leg press with no differences between sexes for the chest press, after controlling for training status [3].

While previous studies have investigated the effects of resistance training experience on the accuracy of ERF, the training status criteria are typically based on years of consistent resistance training in combination with an indicator of muscle strength [5,8]. Experienced compared to novice lifters were found to produce slower velocities when using heavy loads (i.e., 90–100% one repetition maximum—1RM), which was suggested to be due to greater neuromuscular efficiency (i.e., quicker recruitment of high-threshold motor units) [5,8]. It is unknown whether differences in the neuromuscular efficiency between lifters could influence the accuracy of ERF. To date, there is no evidence that muscular performance (e.g., strength, power, endurance) influences the accuracy of ERF in experienced resistance trainers.

Among lifters with similar resistance training experience (quantified in years), it would be expected that a large range of muscle performance abilities would be present. The variability in muscle performance among lifters with similar resistance training experience is the result of a combination of biological, morphological, psychological, and environmental factors [9,10]. Since ERF is a tool that can be used to monitor and prescribe resistance training [2], it is of importance to investigate whether the accuracy of ERF is influenced by muscle performance capabilities, more specifically, whether differences in muscular strength and local muscular endurance (LME) affect the accuracy of ERF. Briefly, LME is defined as the ability to resist muscular fatigue when using a submaximal resistance [11]. Assessment of LME is commonly determined by the maximal number of repetitions to concentric failure when using absolute loads (i.e., a fixed load such as 50kg) or relative loads (i.e., based on % 1RM) [12]. Previous studies suggest that the ability to perform a greater number of repetitions after reporting ERF at a specific time point (e.g., 10th repetition at 70% 1RM) is the main factor negatively affecting accuracy of ERF [3,4]. Therefore, it appears that differences in LME rather than in muscular strength between resistance trainers would influence the accuracy of ERF, when ERF is reported after the completion of a specific number of repetitions with given relative load (% 1RM).

The purpose of this exploratory study was to examine whether muscular strength and LME influences error in ERF (accuracy). It was hypothesized that lifters with greater LME would have lower accuracy of ERF, while muscular strength would not influence accuracy of ERF. A secondary objective of this study was to assess whether muscular strength and LME influences MCV variables, which seems to interact with the accuracy of ERF. It was hypothesized that lifters with greater muscular strength would experience greater MCV loss and achieve the lowest MCV (referred to as MCV_min_) across all repetitions performed during a set.

## 2. Materials and Methods

### 2.1. Research Design

This exploratory study involves analysis of data from a previously published study to examine the effect of muscular strength and LME on accuracy of ERF [13]. Therefore, no power analysis was conducted to identify the sample size needed to detect statistical significance. The participants were separated into two groups based on muscular strength and LME performance for the bench press and squat. After separation of the participants into their respective groups, the variables of interest were analysed to identify whether muscle performance influenced accuracy of ERF and MCV variables. The High-Strength group for the bench press was defined as having a Wilks coefficient ≥70, which was the criteria used by Ormsbee et al. [5] to determined experienced benchers. Based on these criteria, there was an even split of participants in the High-Strength and Low-Strength groups (*n* = 10 for each group). The High-Strength group for the squat was defined as having a Wilks coefficient ≥90, which was the eligibility criteria used by Zourdos et al. [8] to determine experienced squatters. Based on these criteria, there were 11 participants that qualified for the Low-Strength group and 9 participants for the High-Strength group.

Local muscular endurance (LME) was determined by the maximum number of repetitions performed during the first set of each exercise. Since there are no established criteria to determine degrees of LME from a resistance exercise test, the median number of repetitions was considered the cut-off point. For both the bench press and squat, the criteria for High-LME were ≥17 repetitions. Based on these criteria, there was an even split between High-LME and Low-LME groups for the bench press (*n* = 10 in each group), while for the squat, there were 11 participants in the High-LME and 9 participants in the Low-LME groups.

### 2.2. Participants

Twenty males (age 26.3 ± 6.9 years; body mass 82.0 ± 6.0 kg; height 178.0 ± 5.5 cm) participated in this study. The participants had 6.9 ± 4.7 years of resistance training experience. To be eligible to take part in this study, potential participants needed to be male, healthy (absence of musculoskeletal conditions and chronic disease), aged between 18–45 years, have resistance training experience of ≥1 year, and be able to perform the bench press and squat. All participants were informed of the purpose, risks, benefits, and experimental procedures of the study. Prior to commencing the study, all participants signed a written informed consent document. This study was approved by the University of Sydney Human Research Ethics Committee (approval number: 2014/996).

Participants attended the laboratory on three separate occasions. The first visit involved 1RM testing (bench press and squat), the 1RM tests were repeated in the second visit prior to completing a familiarization of the experimental protocol, and the final visit involved completing the experimental session. There was approximately one week in between the familiarization and experimental sessions. The majority of participants completed the familiarization and experimental sessions at a similar time of day (within 2 h for 15 participants), while the time of day for both sessions differed by 3.5–6.5 h for the other participants (due to transport and other commitments). In preparation for each visit, participants were instructed to avoid any strenuous physical activity in the prior 24–48 h, not consume caffeine or pre-workout supplements (2–3 h prior), and not eat within 1 h prior to the visits. Participants were not required to follow a specific diet prior to each visit.

### 2.3. Experimental Session

Prior to commencing the experimental session, participants were educated about using the ERF scale. The memory-anchoring procedure was used to enable participants to relate exercise intensities with the ERF scale [1,3]. As an example, participants were asked to recall times during resistance training sessions when exertion was equal to both ends of the scale (i.e., ‘≤5′ and ’>5’). A warm-up was performed that involved completing approximately 2 sets of 6–10 repetitions (~1-min recovery between sets) at a perceived moderate load for the bench press and squat. Following the warm-up, participants completed 5 sets of 10 repetitions with 70% 1RM. This load was chosen so that participants could perform approximately 20 repetitions to failure for the exercises [14], hence, allowing for a broad range of ERF responses (i.e., 0–10 repetitions to failure). A lighter load was also considered to be less specific for a resistance trainer, considering that muscular hypertrophy and strength development are optimized when using ≥65% 1RM [15]. All sets of repetitions involved consecutive eccentric and concentric contractions.

After the concentric phase of the 10th repetition was completed (full elbow and knee extension for the bench press and squat, respectively), participants paused for approximately 5 s and reported their ERF. This protocol has been performed in previous studies investigating ERF [1,3]. The scale was displayed on a piece of paper and was shown to the participants when they were asked to report their responses. After reporting the ERF, participants were instructed to continue performing repetitions to concentric failure, which was referred to as actual repetitions to failure (ARF). To ensure that participants terminated a set at the point of concentric failure, consistent verbal encouragement was provided. There was 5 min recovery in between each set. This amount of recovery was deemed necessary to ensure that most participants could complete at least 10 repetitions for all 5 sets using 70% 1RM and is consistent with previous studies investigating ERF [1,3]. The exercises were performed using a power rack with the safety rails positioned slightly below the range of motion for the eccentric phase to reduce any risks associated with performing the sets to concentric failure. The correct technique was monitored for all repetitions (as per the 1RM testing protocol), and the repetition speed was self-selected, although a controlled speed was emphasized (no ballistic movements). A self-selected cadence was used to allow the sessions to be consistent with the training practices generally used by recreational trainers and due to the greater training volumes and muscle activation that are produced with self-selected cadences [16].

### 2.4. One-Repetition Maximum (1RM)

The 1RM was assessed for the bench press and squat in this order, with a warm-up performed prior to commencing 1RM attempts for each exercise. The warm-up involved a set of 6–10 repetitions using a perceived moderate load and then a set of 2–3 repetitions using a perceived slightly heavier load (1–2 min recovery between sets). After the warm-up, the participants commenced 1RM testing, which determined the heaviest load they could successfully lift (i.e., for a single repetition) for the bench press and squat. If an attempt was successful, the load was increased. If unsuccessful, the load was decreased, and this process continued until the maximum load the participant could successfully lift was determined. The researcher provided strong verbal encouragement to participants during the 1RM attempts, and there was 3–5 min recovery between each attempt. The technique emphasized for the bench press included lowering the barbell to approximately 2.5 cm from the chest and then pressing the barbell vertically until full elbow extension was achieved. Participants did not allow the barbell to touch their chest to ensure that the barbell would not make contact with the safety rails of the power rack (as detailed above) and interfere with the exercise. In preparation for each bench press 1RM attempt, the researcher assisted the participant with lifting the barbell from the rack. The technique emphasized for the back squat involved flexing the knees and hips until the thighs were parallel to the floor on the descent and then ascending to an upright position. An attempt was considered unsuccessful if participants failed to adhere to the advised exercise techniques.

The intraclass correlation coefficient (ICC) and coefficient of variation (CV) was used to assess the reliability of the 1RM testing sessions (separated by an average of 30 days). There was good reliability for the bench press 1RM (ICC = 0.95; CV = 2.9%) and squat 1RM (ICC = 0.97; CV = 3.1%) [17,18]. The loading for the familiarization and experimental sessions was based on the best 1RM result.

### 2.5. Local Muscular Endurance (LME)

As previously mentioned, LME was assessed from the maximum number of repetitions completed during the first set of the bench press and squat. The 70% 1RM load used during the sets had previously been used to assess LME [19,20]. Although there was a brief pause in between the 10th and 11th repetitions, this was consistent between participants and deemed to not dramatically influence performance. The best result for LME from the familiarization and experimental sessions was used.

### 2.6. Velocity Measures

The GymAware linear position transducer (Kinetic Performance Technology, Mitchell, Australia) was used to assess barbell MCV during all sets. This device has been shown to be valid and reliable for assessing kinetic and kinematic outputs [21]. Calculation of MCV was determined by dividing barbell displacement by concentric phase duration (i.e., from initial vertical displacement to movement cessation). The MCV of repetitions 1 and 2 was averaged (MCV_rep_1,2_) and the MCV of repetitions 9 and 10 (i.e., the two repetitions prior to providing the ERF) were also averaged (MCV_rep_9,10_). Loss of MCV was calculated across a set prior to the reporting of ERF by (MCV_rep_9,10_ minus MCV_rep_1,2_) divided by MCV_rep_1,2_. The minimal MCV (MCV_min_) was determined as the lowest MCV across repetitions in each set. The proximity to MCV_min_ was calculated via the MCV of repetition 10 minus MCV_min_.

### 2.7. Statistical Analyses

Actual repetitions to failure (ARF) > 10 were removed from the analysis of data to avoid misrepresenting the error in ERF at the highest end of the 0–10 ERF scale. Additionally, data were only analysed from sets where participants could perform at least 10 repetitions. The absolute difference between ERF and ARF for each set determined the error in ERF. One-way ANOVA was used to examine differences between groups for all variables. Estimates of effect size (ES) were calculated using Hedges’ *g* (mean difference divided by pooled weighted standard deviation). ESs were interpreted as: <0.2, 0.2 to 0.6, >0.6 to 1.2, >1.2 to 2.0 and >4.0 for small, moderate, large, very large and extremely large effects, respectively [22]. Reliability between the familiarization and experimental sessions was examined for all variables using ICC. The results of the ICC were interpreted as <0.5 poor, 0.5–0.75 moderate, >0.75–0.9 good, and >0.9 excellent reliability [18]. Statistical analyses were performed using SPSS version 28.0 for Windows (IBM Corp., Armonk, NY, USA). Data are reported as means ± standard deviation (SD) with *p* < 0.05 considered statistically significant.

## 3. Results

### 3.1. Characteristics of the Groups

The characteristics of the muscular strength and LME groups is provided in Table 1. For both the bench press and squat, the High-Strength group had significantly greater 1RM (kg) (*p* < 0.001, ES ≥ 1.55), 1RM (kg/kg BM) (*p* < 0.001, ES ≥ 1.60), and Wilks score (*p* < 0.001, ES ≥ 1.71). There were no other differences in the characteristics between the High-Strength and Low-Strength groups. The High-LME compared to Low-LME group performed a significantly higher number of repetitions to failure for the bench press and squat at 70%1RM (*p* < 0.001, ES ≥ 2.04). There were no other differences between groups for the remaining characteristics such as resistance training experience. Data from 96% of sets for the bench press and 91% of sets for the squat were used to analyse ERF, ARF, error in ERF and the MCV variables.

### 3.2. High Strength versus Low Strength

There were no differences between groups for ERF, ARF and error in ERF for the bench press (Table 2). The MCV_min_ was greater in the High-Strength compared to Low-Strength group during set 3 for the bench press, which demonstrated a large ES (*p* = 0.02, ES = −0.96) (Table 3). The Low-Strength group had greater proximity to MCV_min_ when reporting ERF at set 3 with a large ES (*p* = 0.04, ES = 0.96). As for the other bench press MCV variables, there were no differences between groups.

In set 1 of the squat, there was a very large ES indicating greater error in ERF for the Low-Strength compared to High-Strength group; however, it failed to reach statistical significance (*p* = 0.06, ES = 1.63) (Table 2). For set 2, large effect sizes were found in favour of greater MCV_min_ for the High-Strength group (ES = −0.96) and greater proximity to MCV_min_ for the Low-Strength group (ES = 1.15); however, statistical significance was not reached. No differences between groups were found for ERF, ARF, error in ERF and MCV variables for the squat (Table 2 and Table 3).

### 3.3. High-LME versus Low-LME

**Figure 1:**
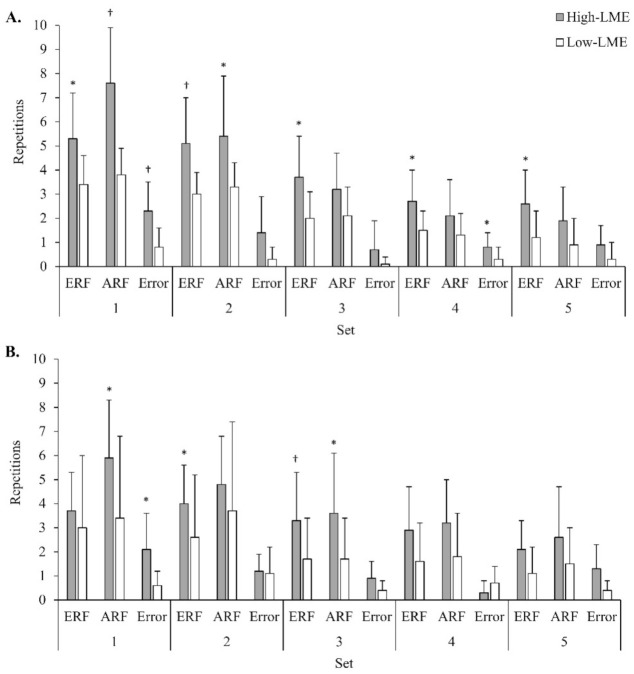
Comparison of repetitions to failure (estimated and actual) and error in estimated repetitions to failure per set between high and low local muscular endurance groups. (**A**) Bench press (High-LME *n* = 11, Low-LME *n* = 9); (**B**) Squat (High-LME *n* = 10; Low-LME *n* = 10). LME, local muscular endurance; ERF, estimated repetitions to failure; ARF, actual repetitions to failure; error = ERF minus ARF (error in ERF) * Statistical significance at *p* <0.05. † Statistical significance at *p* < 0.01. Data presented as mean ± SD.

The High-LME compared to Low-LME group had greater ERF for all five sets of the bench press (*p* < 0.05), and the ES between groups was large for all sets (ES = −0.88 to −1.06) (Figure 1A). The ARF was greater in the High-LME group for the bench press for set 1 (*p* < 0.001, ES = −1.58 (very large)) and set 2 (*p* = 0.04, ES = −0.80 (large)). For sets 3–5 there were moderate to large ESs in favour of greater ARF for the High-LME group (ES = −0.50 to −0.70), although statistical significance was not reached. Error in ERF was greater for the High-LME compared to Low-LME group for the bench press in set 1 (*p* < 0.01, ES = −1.19 (large)) and in set 4 (*p* = 0.04, ES = −0.80 (large)). For sets 2, 3 and 5, there were moderate to large ESs in favour of greater error in ERF for the High-LME group (ES = −0.48 to −0.71), although statistical significance was not reached. The High-LME group had a greater proximity to MCV_min_ when reporting ERF at set 1 (*p* = 0.01), and the ES between groups was large (ES = −1.07) (Table 4). No other significant differences between groups were found for the bench press MCV variables across sets.

The ERF was greater for the High-LME compared to Low-LME group for the squat in set 2 (*p* = 0.04, ES = −0.84 (large)) and set 3 (*p* = 0.03, ES = −0.77 (large)) (Figure 1B) For sets 1, 4, and 5 there were moderate to large ESs in favour of greater ERF for the High-LME group (ES = −0.42 to −0.79), but statistical significance was not reached. The ARF was greater in the High-LME group for the squat in set 1 (*p* = 0.01, ES = −0.99 (large)) and in set 3 (*p* = 0.04, ES = −0.73 (large)). Moderate to large ESs in favour of greater ARF for the High-LME were found in sets 2, 4, and 5 (ES = −0.50 to −0.74), although statistical significance was not reached. Error in ERF was greater for the High-LME compared to Low-LME group for the squat in set 1 and demonstrated a large ES (*p* = 0.01, ES = −0.95). Large ESs in favour of greater error in ERF for the High-LME group was found in set 3 (ES = −0.68) and for the Low-LME group in set 4 (ES = 0.85), although statistical significance was not reached. There were no differences between groups for the MCV variables (Table 4).

### 3.4. Reliability between Familiarization and Experimental Sessions

For the bench press, there was good reliability across sets for ERF (ICC = 0.86) and ARF (ICC = 0.83). Moderate reliability was found for MCV loss (ICC = 0.65) and proximity to MCV_min_ (ICC = 0.56). There was poor reliability for error in ERF (ICC = 0.49) and MCV_min_ (ICC = 0.23) between sessions across sets for the bench press. For the squat, there was moderate reliability found for ERF (ICC = 0.72), ARF (ICC = 0.57) and proximity to MCV_min_ (ICC = 0.55). Poor reliability across sets of the squat was found for MCV_min_ (ICC = 0.34), error in ERF (ICC = 0.26) and MCV loss (ICC = 0.10).

## 4. Discussion

This exploratory study investigated whether muscular strength and LME influences the accuracy in ERF and changes in movement velocity when resistance training. Differences in LME influenced the accuracy of ERF during the first and fourth set of the bench press and first set of the squat, with the Low-LME group having a lower error in ERF. For the bench press, ERF was greater for all sets, and ARF was greater for the first two sets in the High-LME group. Greater ERF and ARF was found for two of the five sets in the High-LME group for the squat. For the bench press, greater proximity to MCV_min_ was observed in the initial set for the High-LME group, but there were no other differences between the LME groups for the other MCV variables. Strength levels did not affect ERF, ARF, and error in ERF for the bench press and squat. For the third set of the bench press, the High-Strength group showed greater MCV_min_ and the Low-Strength group greater proximity to MCV_min_. The findings from this study indicate that LME influences the accuracy of ERF during the initial set of the bench press and squat as well as a latter set for the bench press.

The present study showed that muscle strength may not be a sensitive indicator of the ability to accurately estimate repetitions to failure when resistance training. Typically, muscle strength together with years of resistance training is used to provide an indication of resistance training experience [5,8]. However, as indicated in the present study, the type of muscle performance (i.e., strength or endurance) used to characterize a group should be specific to the nature of the study or intervention. Since our study involved performing multiple sets of resistance exercises to concentric failure with a load of 70% 1RM, performance was more likely to be dependent on muscle endurance abilities rather than their 1RM (e.g., fatigue index) [23]. When comparing the High-Strength and Low-Strength groups, the ARF for all sets of the bench press and squat were not statistically different. In contrast, greater ARF was observed for two sets of the bench press and squat in favour of the High-LME group. For the remaining three sets, moderate to large ESs in favour of greater ARF for High-LME were found, suggesting that the endurance capabilities of the High-LME group may have been more pronounced across sets with a larger sample size.

The accuracy of ERF is highly dependent on the proximity to concentric failure [3]. However, during some sets where differences between groups in ARF were found, error in ERF between groups was not different, consequently suggesting that proximity to concentric failure may not always predict the accuracy of ERF. As mentioned above, the sample size for the present study was most likely underpowered since it was exploratory, which may explain the inconsistent findings concerning ARF and error in ERF. As an example, moderate to large ESs in favour of greater error in ERF in the High-LME group was found for both exercises. For future study investigating the effect of LME on accuracy in ERF, a sample size of 64 (32 participants per group) would be needed to achieve 80% power at *p* < 0.05 (2-sided). This is based on the present study data of error in ERF that showed large ESs (non-significant) in favour of LME (sets 2 and 5 for bench press; sets 3 and 5 for squat).

When a set of resistance exercise is performed to concentric failure, there are reductions in MCV due to fatigue [6]. This change in MCV is the result of a decline in muscle fibre shortening speed, relaxation time, and force production [24]. The MCV of the last repetition of a set prior to concentric failure, which is referred to in the present study as MCV_min_, has been shown to match MCV during 1RM performance [25]. Potentially, a lifter may utilize any noticeable change in MCV during a set to assist with ERF. For the bench press, a greater proximity to MCV_min_ when reporting ERF was found for the Low-Strength group in set 3. However, no differences were observed between strength groups for error in ERF for the bench press, suggesting proximity to MCV_min_ when reporting ERF may not affect the accuracy of ERF. In contrast to the strength group findings, when the high and low LME groups were compared, the proximity to MCV_min_ when reporting ERF during the bench press in set 1 appeared to influence the accuracy. The High-LME group had greater proximity to MCV_min_ when reporting ERF as well as greater error in ERF during the bench press in set 1. Therefore, less accurate ERF for the bench press may occur during early sets in lifters with greater LME when there is greater proximity to MCV_min_.

Since previous studies have reported greater accuracy of ERF for upper body compared with lower-body resistance exercises [1,3], it seems plausible that a lifter may be able to better utilize changes in MCV to guide their ERF. This would consist of an interplay of afferent and efferent feedback to determine the number of repetitions possible during a set [26]. Compared to the lower limbs, the upper limbs are involved in more precise movements in everyday activities and possess a higher sensory organ density [27]. While strong relationships have been found between MCV and RPE [5,8], only weak relationships have been shown between MCV and error in ERF [13]. However, a lifter with greater LME may have better pain tolerance that could desensitize them to exertional signals which has been purported to influence the reporting of RPE when resistance training [28]. As an example, following repeated exposure to high intensity interval training, which is known to cause discomfort, increases in pain tolerance have been observed [29]. It should also be highlighted that the accuracy of ERF might best be assessed on an individualized basis. García-Ramos et al. [30] showed that generalized group equations for ERF from the fastest MCV were not acceptable due to the between-subjects variability (CV >10%). Therefore, numerous factors including strength, LME, and other training-related factors should be considered when examining determinants of error in ERF.

Some limitations should be acknowledged when interpreting the present study findings. As mentioned earlier, the sample size was relatively small and most likely influenced the number of statistically significant findings. The present study could have gathered further information concerning the training history of participants (i.e., resistance exercise prescription) which would have allowed more in-depth exploration of factors influencing the accuracy of ERF. Future research on the topic of factors that influence the accuracy of ERF should consider incorporating a wider range of physiological, psychological and training-related data. Additionally, the relative strength of the participants in the High-Strength group was a little lower compared to similarly aged powerlifters for the bench press (i.e., 1.3 versus 1.6 × body weight for bench press, respectively) and squat (1.7 versus 2.3 × body weight for squat, respectively) [31]. The findings of the present study may differ in a cohort of stronger resistance trainers. As with all studies investigating ERF, it is possible that participants could have terminated sets once reaching their ERF, thus biasing the accuracy of ERF. Furthermore, it is also possible that the reporting of ERF may have influenced the ARF, e.g., reporting an ERF of three repetitions may have consciously inhibited a participant from striving to complete an additional 4–5 repetitions (i.e., 4–5 ARF). Through consistent verbal encouragement throughout sets, we believe that participants terminated sets at concentric failure. However, it is unknown whether the reporting of ERF influenced the ARF performed by a participant. Finally, there was poor reliability between the familiarization and experimental sessions for a number of variables, including error in ERF. Future studies are required to further examine the reliability of error in ERF between sessions and factors that may contribute towards fluctuations in the accuracy of ERF between sessions.

## 5. Conclusions

The findings of the present study suggest that male resistance trainers with greater LME compared to strength are less accurate when reporting ERF during initial sets of the bench press and squat. It seems plausible that a similar trend would exist during latter sets as observed in the fourth set of the bench press in the present study, and future studies with larger sample sizes are warranted to confirm this hypothesis. When planning to use the ERF scale during resistance training, coaches and trainers need to consider assessing LME. With lifters that are able to perform a greater number of repetitions at a specific %1RM, hence indicating greater LME, the error in ERF will probably be greater when a designated number of repetitions (not to concentric failure) is prescribed. This is due to greater proximity to concentric failure which is known to negatively influence the accuracy in ERF. Utilizing devices to measure barbell velocity such as a linear position transducer would be ideal to gauge proximity to concentric failure for barbell exercises and may assist with confirming the accuracy of ERF.

## Figures and Tables

**Table 1:** Characteristics of muscular strength and local muscular endurance groups.

Muscular Strength						
	Bench Press			Squat		
	High (*n* = 10)	Low (*n* = 10)	ES	High (*n* = 9)	Low (*n* = 11)	ES
Age (years)	26.2 ± 4.8	26.4 ± 8.8	0.04	26.0 ± 6.5	26.5 ± 7.5	0.07
Body mass (kg)	82.9 ± 6.7	81.1 ± 5.4	−0.26	80.6 ± 4.4	83.1 ± 7.0	0.54
Height (cm)	176.8 ± 3.7	179.8 ± 6.4	0.78	176.9 ± 4.3	178.8 ± 6.4	0.42
RT experience (years)	7.5 ± 4.4	6.3 ± 5.1	−0.26	7.0 ± 5.6	6.8 ± 4.0	−0.03
1RM (kg)	112.5 ± 10.1 ^†^	92.3 ± 5.3	−1.92	155.6 ± 23.0 ^†^	118.4 ± 12.8	−1.55
1RM (kg/kg BM)	1.4 ± 0.1 ^†^	1.1 ± 0.1	−2.87	1.9 ± 0.3 ^†^	1.4 ± 0.2	−1.60
Wilks score	75.4 ± 5.9 ^†^	62.8 ± 4.6	−2.05	105.8 ± 14.7 ^†^	79.5 ± 9.6	−1.71
**Local Muscular Endurance**						
	**Bench Press**			**Squat**		
	**High** **(*n* = 11)**	**Low** **(*n* = 9)**	**ES**	**High** **(*n* = 10)**	**Low** **(*n* = 10)**	**ES**
Age (years)	25.9 ± 7.9	26.8 ± 5.8	0.11	26.8 ± 7.9	25.8 ± 6.1	−0.12
Body mass (kg)	83.4 ± 7.1	80.2 ± 4.1	−0.43	84.1 ± 6.8	79.9 ± 4.5	−0.59
Height (cm)	178.4 ± 5.3	177.5 ± 5.9	−0.16	178.8 ± 4.7	177.1 ± 6.3	−0.35
RT experience (years)	6.5 ± 4.2	7.3 ± 5.4	0.18	6.7 ± 4.0	7.2 ± 5.4	0.12
Max rep at 70% 1RM	19.1 ± 1.8 ^†^	14.7 ± 0.7	−2.34	19.4 ± 2.4 ^†^	14.3 ± 1.2	−2.04

BM, body mass; ES, effect size; Max rep at 70% 1RM, maximal repetitions at 70% 1RM. Muscular strength, High group criteria Wilks score of ≥70 for bench press and ≥90 for squat. Local muscular endurance, High group criteria was ≥17 repetitions to failure for each exercise. ^†^ Significant difference at *p* < 0.001. Data presented as mean ± SD.

**Table 2:** Comparison of repetitions to failure (estimated and actual) and error in estimated repetitions to failure per set between high and low strength groups for the bench press and squat.

		Bench Press						Squat					
Set	Group	ERF	ES	ARF	ES	Error in ERF	ES	ERF	ES	ARF	ES	Error in ERF	ES
1	High	4.3 ± 2.2	0.09	5.6 ± 2.8	0.14	1.2 ± 1.0	0.67	3.1 ± 1.4	0.27	3.8 ± 1.8	0.69	0.7 ± 0.7	1.63
	Low	4.5 ± 1.6		6.0 ± 2.6		1.9 ± 1.4		3.5 ± 0.8		5.1 ± 2.4		1.9 ± 1.6	
2	High	4.3 ± 2.4	−0.12	4.3 ± 2.8	0.10	1.0 ± 1.6	−0.06	3.3 ± 1.7	0.07	4.1 ± 2.9	−0.06	1.2 ± 1.6	−0.06
	Low	4.0 ± 1.2		4.6 ± 1.6		0.8 ± 0.9		3.2 ± 3.2		4.3 ± 1.7		1.1 ± 0.9	
3	High	3.2 ± 2.2	−0.22	2.7 ± 1.9	0	0.7 ± 1.3	0.24	2.3 ± 1.2	0.30	2.3 ± 1.9	−0.11	0.7 ± 0.9	−0.02
	Low	2.7 ± 0.9		2.7 ± 0.8		0.2 ± 0.4		2.6 ± 2.1		2.9 ± 2.4		0.6 ± 0.7	
4	High	2.4 ± 1.6	−0.24	1.9 ± 1.6	−0.18	0.5 ± 0.7	0.27	2.1 ± 1.2	0.24	2.5 ± 1.5	0.06	0.4 ± 0.7	0.27
	Low	2.0 ± 0.7		1.6 ± 0.9		0.7 ± 0.5		2.4 ± 1.9		2.6 ± 2.2		0.6 ± 0.7	
5	High	2.0 ± 1.8	−0.11	1.8 ± 1.5	−0.57	0.4 ± 0.7	0.68	2.0 ± 1.2	−0.55	2.8 ± 1.9	−0.70	0.8 ± 0.9	0.11
	Low	1.8 ± 0.9		0.9 ± 0.6		0.9 ± 0.8		1.3 ± 1.0		1.4 ± 1.6		0.9 ± 1.1	

RPE, rating of perceived exertion; ERF, estimated repetitions to failure; ARF, actual repetitions to failure; ES, effect size. Muscular strength, bench press (High *n* = 10, Low *n* = 10); squat (High *n* = 9; Low *n* = 11). Data presented as mean ± SD.

**Table 3:** Comparison of mean concentric velocity variables across sets between high and low strength groups for the bench press and squat.

Bench Press								Squat					
Set	Group	MCV Loss	ES	MCV_min_	ES	Proximity to MCV_min_	ES	MCV Loss	ES	MCV_min_	ES	Proximity to MCV_min_	ES
1	High	26.5 ± 11.7	−0.15	0.24 ± 0.06	−0.64	0.17 ± 0.10	−0.10	18.0 ± 11.2	0.58	0.38 ± 0.08	−0.47	0.07 ± 0.07	0.54
	Low	24.7 ± 8.6		0.20 ± 0.05		0.16 ± 0.07		24.8 ± 6.1		0.34 ± 0.11		0.11 ± 0.10	
2	High	25.8 ± 16.7	0.02	0.23 ± 0.06	−0.32	0.14 ± 0.08	0.12	17.7 ± 9.9	0.43	0.39 ± 0.07	−0.96	0.07 ± 0.05	1.15
	Low	26.1 ± 10.5		0.21 ± 0.04		0.15 ± 0.08		22.2 ± 11.6		0.32 ± 0.09		0.13 ± 0.10	
3	High	35.8 ± 9.0	−0.35	0.23 ± 0.05 *	−0.96	0.08 ± 0.05 *	0.96	16.0 ± 17.1	0.57	0.35 ± 0.11	−0.09	0.10 ± 0.06	−0.16
	Low	32.5 ± 9.0		0.18 ± 0.04		0.13 ± 0.06		26.1 ± 12.1		0.34 ± 0.09		0.09 ± 0.08	
4	High	29.3 ± 20.6	0.51	0.25 ± 0.08	−0.48	0.08 ± 0.09	−0.11	21.6 ± 13.5	0.69	0.36 ± 0.11	−0.52	0.09 ± 0.08	0
	Low	40.3 ± 8.8		0.21 ± 0.08		0.07 ± 0.08		31.4 ± 18.5		0.30 ± 0.10		0.09 ± 0.07	
5	High	38.4 ± 15.3	−0.07	0.21 ± 0.04	0	0.09 ± 0.07	−0.68	19.8 ± 11.3	0.34	0.38 ± 0.32	−0.18	0.05 ± 0.05	0.19
	Low	37.2 ± 16.0		0.21 ± 0.03		0.04 ± 0.05		23.9 ± 14.4		0.32 ± 0.07		0.06 ± 0.06	

MCV, mean concentric velocity; Loss of MCV, (MCV_rep_9,10_ minus MCV_rep_1,2_) divided by MCV_rep_1,2_; MCV_min_, minimal mean concentric velocity; Proximity to MCV_min_ calculated by MCV on the 10th repetition minus MCV_min_; ES, effect size. Muscular strength, bench press (High *n* = 10, Low *n* = 10); squat (High *n* = 9; Low *n* = 11). * Statistical significance at *p* < 0.05. Data presented as mean ± SD.

**Table 4:** Comparison of mean concentric velocity variables across sets between participants with high and low local muscular endurance for the bench press and squat.

		Bench Press						Squat					
Set	Group	MCV Loss	ES	MCV_min_	ES	Proximity to MCV_min_	ES	MCV Loss	ES	MCV_min_	ES	Proximity to MCV_min_	ES
1	High	29.8 ± 11.1	−0.70	0.21 ± 0.06	0.48	0.21 ± 0.08 *	−1.07	22.1 ± 11.6	−0.16	0.32 ± 0.10	0.57	0.11 ± 0.10	−0.38
	Low	21.7 ± 7.3		0.24 ± 0.05		0.12 ± 0.07		20.1 ± 8.4		0.38 ± 0.08		0.07 ± 0.08	
2	High	22.4 ± 14.3	0.53	0.21 ± 0.05	0.38	0.17 ± 0.09	−0.64	17.1 ± 8.9	0.60	0.35 ± 0.09	0	0.09 ± 0.09	0.11
	Low	30.3 ± 12.0		0.23 ± 0.06		0.11 ± 0.06		22.7 ± 12.0		0.35 ± 0.09		0.10 ± 0.08	
3	High	30.0 ± 10.2	0.86	0.21 ± 0.04	−0.24	0.12 ± 0.07	−0.41	18.0 ± 14.0	0.49	0.33 ± 0.10	0.19	0.11 ± 0.05	−0.57
	Low	39.2 ± 10.6		0.20 ± 0.07		0.09 ± 0.04		25.1 ± 16.0		0.35 ± 0.10		0.08 ± 0.08	
4	High	34.3 ± 17.3	0.03	0.23 ± 0.09	0	0.07 ± 0.10	0.10	25.3 ± 14.3	0.29	0.30 ± 0.09	0.53	0.09 ± 0.08	0
	Low	34.9 ± 16.9		0.23 ± 0.06		0.08 ± 0.05		29.6 ± 20.0		0.35 ± 0.12		0.09 ± 0.07	
5	High	33.5 ± 18.1	0.46	0.20 ± 0.04	0.48	0.09 ± 0.08	−0.48	21.0 ± 14.7	0.11	0.32 ± 0.07	0.81	0.07 ± 0.06	−0.63
	Low	42.2 ± 10.8		0.22 ± 0.04		0.05 ± 0.05		22.7 ± 11.2		0.38 ± 0.08		0.03 ± 0.04	

MCV, mean concentric velocity; Loss of MCV, (MCV_rep_9,10_ minus MCV_rep_1,2_) divided by MCV_rep_1,2_; MCV_min_, minimal mean concentric velocity; Proximity to MCV_min_ calculated by MCV on the 10th repetition minus MCV_min_; ES, effect size. Local muscular endurance, bench press (High *n* = 11, Low *n* = 9); squat (High *n* = 10; Low *n* = 10). * Statistical significance at *p* < 0.05. Data presented as mean ± SD.

## Data Availability

Not applicable.
